# Quality assessment of diagnostic before-after studies: development of methodology in the context of a systematic review

**DOI:** 10.1186/1471-2288-9-3

**Published:** 2009-01-19

**Authors:** Catherine A Meads, Clare F Davenport

**Affiliations:** 1 Unit of Public Health, Epidemiology and Biostatistics, University of Birmingham, Birmingham, UK

## Abstract

**Background:**

Quality assessment tools for primary studies of test accuracy are relatively well developed, although only one is validated (QUADAS), but very little work has been done to develop tools to quality-assess studies evaluating the impact of diagnostic testing on management of patients (diagnostic or therapeutic yield). The recent draft NICE Guide to the Methods of Technology Appraisal (2007) suggests QUADAS "as a useful starting point for appraising studies that evaluate the sensitivity and specificity of a test" but does not mention how to quality assess diagnostic or therapeutic yield studies, in particular diagnostic before-after studies. In the context of undertaking a rapid systematic review of structural neuroimaging in psychosis for NICE, we describe the modifications that we made to QUADAS, our experience of this in practice and in relation to published theory on diagnostic or therapeutic yield studies.

**Methods:**

The QUADAS tool was assessed for use in the review by two systematic reviewers with in-depth knowledge of the clinical area being reviewed and the types of studies being found in the searches that could answer the clinical question. Modifications were made following discussion as considered appropriate.

**Results:**

Two QUADAS questions were removed altogether and. four additional questions were developed to capture additional quality issues not addressed by QUADAS. However, the developed checklist only partially helped to discern implications of the study designs on the results given.

**Conclusion:**

The division between topic-specific and more generic quality items of relevance to diagnostic before-after studies is important. With more time, further work could have been done to create a better quality assessment tool, for example by incorporating some of the issues mentioned in previous work in this area. This paper is a discussion around quality assessment and is intended to offer insights into the types of issues that should be assessed. A quality assessment tool for diagnostic before-after studies that incorporates items from QUADAS and published theory needs to be further developed and validated.

## Background

The evaluation of diagnostic technologies includes assessment of test accuracy and clinical, process or economic outcomes following testing (see additional file 1)[1]. The impact of a test depends on a variety of factors in addition to test accuracy including; interpretation of tests results, the possibility that the new information does not contribute sufficiently to cross a treatment threshold, clinician awareness of availability of cost-effective treatments, lack of patient access to treatments, acceptability of treatments to patients and the possibility that the patient is already receiving optimal care[2].

Once test results arrive, clinicians use the information to make categorise patients into those with and those without disease, known as the diagnostic yield, and then decisions about treatment required – therapeutic yield[1] (see additional file 1). Diagnostic and therapeutic yield should be considered separately from the accuracy of the test. For example, a test could be 100% sensitive and 100% specific but may not have a high therapeutic yield for a variety of reasons listed in the previous paragraph.

Study designs that may be employed for evaluations of diagnostic and therapeutic yield include randomised controlled trials, or non-randomised experimental or observational studies. Randomised trials may be impractical due to large sample size requirements,[1] the speed of technological advances in diagnostics that risks trial results being obsolete and ethical considerations arising from the potential to deny patients beneficial treatments.

An observational study design which allows for evaluation of diagnostic or therapeutic yield is the diagnostic before-after study, (see additional file 2). For this study design in its most basic form, a group of patients undergo an existing test or battery of tests and the therapeutic strategy is noted, depending on the test results. They then have the new test to be evaluated and any change of diagnosis or treatment strategy is noted and compared. The design can be elaborated to include measurement of test accuracy if the new test is not the reference standard, and assessment of patient outcomes following treatment. Diagnostic before-after studies may be retrospective or prospective in contrast to the temporal relationship traditionally implied by before-after evaluation studies.

Diagnostic before-after studies are subject to a number of limitations[2] such as discrepancies between stated clinical assessment and actual clinical action, and possible subconscious bias about the benefits of the new technology – a clinician may delay making a definitive diagnosis if they know that another test is going to be performed. Also there can be no direct comparison of patient outcomes because all have had the new test. However some of the limitations can be overcome by careful planning and conduct of the study. For example using a prospective design may ameliorate review bias, and independently reviewing pre-and post test clinical assessment and strictly adhering to a study protocol may ameliorate discrepancies between stated clinical assessment and actual clinical action.

Observational studies, such as diagnostic before-after studies, are easier and quicker to conduct than RCTs[3]. In addition it is considered that diagnostic before-after studies tend to be biased in favour of new interventions so when no benefit is found, it is unlikely that a stronger study design on the same question, such as an RCT, will find a benefit[2]. Therefore despite limitations, diagnostic before-after studies may have a role in evaluating therapeutic impact of diagnostic tests.

This paper discusses an example of the use of diagnostic before-after studies to evaluate the effectiveness of structural neuro-imaging in psychosis in the context of undertaking a health technology assessment for the NICE technology appraisals programme in the UK. The systematic review underpinning this methodological paper is published as an HTA monograph[4]. The decision problem for the systematic review underlying this work was to evaluate the added value of structural neuro-imaging with CT or MRI compared to current practice alone. Current practice was defined as any test(s) or investigation(s), or any combination of tests that would be carried out as part of the initial care of a psychotic patient to identify brain lesions in two patient groups – acutely psychotic patients and psychotic patients who are treatment-resistant or deteriorating despite treatment[4]. This decision problem can be conceptualised as a before and after comparison of two diagnostic strategies – current practice only and current practice with CT and/or MRI (see additional file 3) where CT and MRI are considered reference standards for the pathology investigated (target disorders). However, unlike most diagnostic yield studies where a single target condition is investigated, this review had several target conditions i.e. any organic disorder with the potential to cause psychosis as well as any treatable organic condition that may coexist with psychosis; including cerebrovascular accident (CVA), various vascular disorders and brain tumours. The best structural neuroimaging method to determine the presence or absence of these conditions varies depending upon the condition. For example, CT is considered better than MRI for diagnosing calcification, whereas MRI is the gold standard for the diagnosis of space occupying lesions. For the purposes of this review CT and/or MRI were considered reference standard tests for the pathologies being investigated and so additional assessment of test accuracy was not considered a necessary component of included studies. The key question to be answered by the systematic review was whether the addition of neuroimaging would affect diagnostic yield, patient management (therapeutic yield) and ultimately patient outcomes.

In this situation an RCT for diagnostic or therapeutic yield would not be useful because multiple conditions were being sought. If patient outcomes such as health-related quality of life and mortality due to undetected treatable conditions were the outcomes measured, the sample size would need to be massive. Therefore the most likely design that would be found in a systematic review would be a diagnostic before-after study.

In this context it is important to know some information about psychosis in order to appreciate the clinical scenario. In 2005–6 there were 41,600 NHS finished episodes and 2,617,500 bed days in England due to psychotic illnesses[5]. Psychosis secondary to a brain tumour is rare. The prevalence of brain tumours in psychiatric patients has been estimated in a review of cross sectional studies of prevalent cases to be approximately 1.2% (using CT scan). However this does not distinguish between psychotic patients with coincidental brain tumours and patients with brain tumours causing their psychotic symptoms[6]. Psychotic patients can develop additional pathology at any time during their life. Structural neuroimaging (MRI and CT scanning) allows non-invasive visualisation of anatomical structure of the brain in order to assist in the diagnosis of intracranial pathology. As it has been estimated that between 4.3–10% of patients have psychological reactions sufficiently severe to require that MRI has to be modified, postponed or cancelled, it is important to know whether subjecting psychotic patients to this procedure is clinically warranted[7].

When conducting the systematic review, we discovered that there was no existing quality assessment tool for diagnostic before-after studies. Therefore, we had to modify a validated quality assessment tool for diagnostic accuracy studies. We describe the modifications that we made to the QUADAS[8] tool in relation to published theory on diagnostic or therapeutic yield studies[2,3] and our experience of using the modified tool in practice.

Standard systematic review methods were used to find suitable studies to answer the clinical question. The inclusion criteria were any design that gave diagnostic or therapeutic yield, including prospective or retrospective diagnostic before-after studies, reporting the additional diagnostic benefit of structural MRI, CT or combinations of these in patients with psychosis compared to any current standard practice of diagnostic workup without structural neuroimaging. An added complication was whether there were any symptoms and signs of a space occupying lesion or not in patients in the included studies. In the included studies, diagnostic tests conducted before or in addition to structural neuroimaging were often not detailed well but, when described, were a variety of medical and psychiatric history, physical and neurological examinations, biochemical tests, blood tests, toxicological screens, mental state examinations, EEG and psychiatric rating scales. Only studies reporting clinically relevant outcomes were included in the review, such as the proportion of patients with scans identifying pathology that would influence patient treatment (therapeutic yield) and patient outcomes that were not suspected from history and/or physical examination.

Standard systematic review methods include quality assessment of included studies. Quality assessment tools for primary studies of test accuracy are relatively well developed,[8] although only one is validated – QUADAS[8]. Recent draft NICE Guide to the Methods of Technology Appraisal (2007) suggests the QUADAS quality assessment tool "as a useful starting point for appraising studies that evaluate the sensitivity and specificity of a test" but no guidance is provided on quality assessment of diagnostic before-after studies. This lack of a validated quality assessment tool appears not to have been noticed up to now, perhaps because there are very few systematic reviews of diagnostic or therapeutic yield studies. However, it is likely that in the future NICE will be appraising more devices and diagnostic tests (Personal Communication, Carole Longson, NICE, December 2008).

## Methods

A pragmatic decision was made to use QUADAS and adapt it for diagnostic before-after studies. The fourteen QUADAS questions (see table 1) were assessed by two experienced systematic reviewers independently for appropriateness, ease of use and whether the tool would assess the desired qualities and have discriminant ability. This was assessed qualitatively using individual judgement as there were no objective criteria to use. The two reviewers then met to agree which questions to use, how to adapt them and whether additional questions were required. The adapted checklist was piloted on a selection of studies to dermine whether it had reasonable performance characteristics to distinguish between studies, prior to full data extraction and quality assessment.

**Table 1:** Original QUADAS items and additional questions used in the neuroimaging review.

Original QUADAS item	Additions for the structural neuroimaging review
1. Was the spectrum of patients representative of the patients who will receive the test in practice?	

2. Were selection criteria clearly described?	A. Were patients recruited consecutively?

3. Is the reference standard likely to correctly classify the target condition?	Removed for the neuro-imaging review

4. Is the time period between reference standard and index test short enough to be reasonably sure that the target condition did not change between the two tests?	

5. Did the whole sample or a random selection of the sample, receive verification using a reference standard of diagnosis?	

6. Did patients receive the same reference standard regardless of the index test result?	

7. Was the reference standard independent of the index test (the index test did not form part of the reference standard)?	Removed for the neuro-imaging review

8. Was the execution of the index test described in sufficient detail to permit replication of the test?	

9. Was the execution of the reference standard described in sufficient detail to permit replication of the test?	D. Who performed the clinical evaluation and image analysis?

10. Were the index test results interpreted without knowledge of the results of the reference standard?	C. Was the study and/or collection of clinical variables conducted prospectively?

11. Were the reference standard results interpreted without knowledge of the results of the index test?	

12. Were the same clinical data available when test results were interpreted as would be available when the test is used in practice?	

13. Were uninterpretatable/intermediate test results reported?	

14. Were withdrawals from the study explained?	B What was the explanation for patients who did not receive CT or MRI?

*Index test – tests before MRI/CT, Reference test – MRI/CT

For the purpose of this quality assessment the 'before' diagnostic strategy was considered the index test as referred to in QUADAS and the 'after' diagnostic strategy included CT/MRI and was considered to be a reference standard for structural organic pathology where a reference standard is defined as the best test practically available approximating to a final diagnosis.

## Results

The original QUADAS checklist and the modified checklist used for quality assessment in the technology appraisal can be seen in Table 1. The changes from the original QUADAS checklist are described and explained below.

Two items were deleted altogether from the original QUADAS checklist – item 3 and item 7. Item 3 ("Is the reference standard likely to classify the target condition correctly?") was considered redundant because it was presumed in all cases that the reference test (CT or MRI) would classify the target condition correctly. All included studies had to use CT or MRI so Item 3 would not distinguish one study from another within the systematic review.

Item 7 ("Was the reference standard independent of the index test (i.e. the index test did not form part of the reference standard)?") was also considered redundant because in diagnostic before – after tests the 'after' diagnostic strategy (referred to here as the reference test) necessarily incorporated the 'before' component (referred to here as the index test), ie patients would not get CT/MRI alone.

In addition it was felt necessary to add four additional questions to the QUADAS checklist: These extra questions were developed using an established quality assessment development guide[9].

### A. Were patients recruited consecutively?

This question was added because items 1 and 2 on the QUADAS checklist refer to issues of sample selection but consecutive recruitment is not explicitly mentioned. Consecutive recruitment is important to prevent bias in the selection of patients going into the study.

### B. What was the explanation for patients who did not receive CT or MRI?

This question was added because although Item 14 on the QUADAS checklist is concerned with whether an explanation for withdrawals from the study was given, it does not include documentation of reasons. For this review it was important to distinguish between the studies where patients were withdrawn at the start of the study because they refused consent and those studies with patients who had consented but CT/MRI results were not presented, possibly because the scan results had been lost. It also highlighted studies where patients could not tolerate the procedure, particularly for MRI scanning.

### C. Was the study and/or collection of clinical variables conducted prospectively?

QUADAS does not address study design explicitly although items 10 and 11 capture bias that may be introduced by a retrospective versus a prospective study design. It was decided for the purposes of this review that it was important to know whether the study was a retrospective chart review with possible bias in the selection of patients, results of scans and treatment decisions.

### D. Who performed the clinical evaluation and image analysis?

Items 8 and 9 on the QUADAS checklist are concerned with replication of the index and reference test. However details of their conduct are not required for completion of the checklist. For the neuro-imaging review test conduct was important because of the subjective nature of interpretation. It is likely that there might be different results of the scan was read by a radiologist compared to a psychiatrist.

In summary, although QUADAS may implicitly address most of these issues of validity, for the purposes of this review of diagnostic before-after studies we decided it was necessary to record the presence of absence of these characteristics explicitly.

Application of the checklist to the 24 diagnostic before-after studies included in the systematic review was successful in identifying differences in study conduct and quality[8]. Following tabulation of quality criteria in the systematic review, using the modified QUADAS checklist, possible threats to study validity were used to guide interpretation of results and future research recommendations. However, the developed checklist did not lead to that much greater insight into the relationship between potential threats to study validity identified by the checklist and the direction of results of the studies.

## Discussion

The HTA found that structural neuroimaging identified little to influence patient management that was not suspected from medical history and/or physical examination. NICE guidance from this appraisal states the following "Structural neuroimaging, using methods called magnetic resonance imaging (MRI) or computed axial tomography (CT) scanning, is not recommended for use routinely to examine all people who have had a first episode of psychosis[10]."

Quality assessment using our checklist helped to assess the included studies and drive report conclusions. It helped to identify the extent of poor reporting of most of the studies and drew attention to important omissions. For example, it helped focus on the details of 'index test' assessments. These were often poorly reported in the included studies and it was frequently not clear what other assessments had been made in addition to scanning. Because of the checklist we were able to offer a consistent and transparent assessment of quality of the included studies.

However, our checklist did not allow us to make a clear distinction between test results and the contribution they made to the subsequent diagnosis. For example, some of the studies appeared to have interpreted scan results without knowledge of other assessments, some had other results available when interpreting the scan results but for most studies it was not clear whether the scan results had been interpreted without knowledge of the results of other assessments. In most cases it was not possible to tell whether the same clinical results were available when test results were interpreted as would be available in practice. It was also unclear in most studies how the scan results had changed diagnosis. On the one hand, this issue is to do with blinding of test interpretation, but on the other hand, it is about the complex and often subconscious nature in which diagnoses are made[11] and how we assess the contribution of a test result to obtain a final diagnosis.

We did not define acceptable variation in the definition of the reference standard beforehand. As a result, there was some confusion as to whether the reference standard in one study was similar enough to that in other studies for narrative synthesis. Subsequently it has made us think much more clearly about how we would define a reference standard for these types of studies in the future. For example in the review discussed here the reference standard could have been defined as one or more of the following:

• Medical history, physical examination, CT and/or MRI

• Medical history, physical examination, laboratory tests, CT and/or MRI

• Medical history, physical examination, laboratory tests, EEG, CT and/or MRI

This is an area where an improved checklist with a more tightly worded question set would be more useful.

Our checklist did not allow us to capture sufficient detail about patient spectrum. In this clinical area, the following difficulties with patient spectrum became apparent:

• The date of presentation of the first episode does not usually coincide with the onset of the condition because the person could have had psychotic symptoms for years without presenting to a health professional and often psychosis has a gradual onset

• The duration of untreated psychosis is important because it predicts response to treatment[12]

• A first episode could continue for ten years or more without remission, even when the patient is having treatment[13]

• The reason for referral of the patients into the study could have been for clinical reasons, routine scanning on admission or for research purpose

• The setting of the study (primary, secondary or tertiary care) might be expected to yield different results

It is likely that the prevalence of space-occupying lesions in each group would be different. A better checklist would have provided more detail about the spectrum of included patients.

The examples above illustrate that there is an overlap between study characteristics and quality of the study and that documentation of study characteristics is an important element of quality assessment. Other issues that were not assessed by our checklist included whether there was an independent review of the diagnostic decisions and/or treatment decisions made for each patient and whether there was an appropriate sample size calculation

## Conclusion

Evaluation of test accuracy is a component of test evaluation distinct from diagnostic yield, therapeutic yield and patient outcomes. We did not assess the test accuracy of the new tests since CT and MRI scanning are accepted reference standards. In situations where the after test is not an accepted reference standard, assessment of test accuracy would also be required. In fact, it is known that CT does not have 100% sensitivity and specificity[2] so there may have been some misclassification for false positives and false negatives.

Guyatt[2] introduced the role of before and after studies and their strengths and limitations. Some of the issues he raised overlap to some extent with items on the QUADAS checklist (see Table 2), even though QUADAS is aimed at diagnostic accuracy as opposed to diagnostic and therapeutic yield studies. It can be seen from this table that there are additional issues that Guyatt considered. However, as illustrated above, QUADAS does not capture all pertinent issues either. Our attempts to modify QUADAS addressed some but not all of these. The aim of this paper is to start a process whereby an appropriate quality assessment checklist can be developed and validated according to established and accepted development procedures.

**Table 2:** Comparison of QUADAS items^8 ^and validity issues raised by Guyatt.^2^*

QUADAS item (outcome = test accuracy)	Methodological issues raised by Guyatt (outcome = therapeutic impact)
1. Was the spectrum of patients representative of the patients who will receive the test in practice?	The specific clinical indication for performing the test should be clear.

2. Were selection criteria clearly described?	Bias may be introduced if the sample does not comprise consecutive patients presenting with a specific clinical problem

3. Is the reference standard likely to correctly classify the target condition?	Not all before after studies evaluate a reference standard as the after test. Where the after test is not a recognised reference standard for the target disease full delineation of the impact of test errors requires evaluation of all study subjects with a reference standard.

4. Is the time period between reference standard and index test short enough to be reasonably sure that the target condition did not change between the two tests?	Not addressed by Guyatt

5. Did the whole sample or a random selection of the sample, receive verification using a reference standard of diagnosis?	Not all before after studies evaluate a reference standard as the after test. Where the after test is not a recognised reference standard for the target disease full delineation of the impact of test errors requires evaluation of all study subjects with a reference standard.

6. Did patients receive the same reference standard regardless of the index test result?	Not addressed by Guyatt

7. Was the reference standard independent of the index test (the index test did not form part of the reference standard)?	Before after studies are concerned with evaluating the value of adding a test. Consequently the results of the before test are usually known at the time of interpreting the after test results. This item is therefore redundant for diagnostic before-after studies.

8. Was the execution of the index test described in sufficient detail to permit replication of the test?	Whether a diagnostic technology has therapeutic impact might depend on the physician using the test.

9. Was the execution of the reference standard described in sufficient detail to permit replication of the test?	

10. Were the index test results interpreted without knowledge of the results of the reference standard?	To evaluate the technology's contribution to change in therapy therapeutic plans based on the 'before test' must be elicited before knowledge of the 'after test' results.

11. Were the reference standard results interpreted without knowledge of the results of the index test?	Before after studies are concerned with evaluating the value of adding a test. Consequently the results of the before test are usually known at the time of interpreting the after test results. However therapeutic plans based on the before test results should not be known at the time of making therapeutic plans based on a combination of the before and after test results. Before-after studies can be strengthened by an independent review of the after test's contribution to therapeutic decisions relative to the before test.

12. Were the same clinical data available when test results were interpreted as would be available when the test is used in practice?	The addition of a test may have appeared to have had an impact because of an incomplete pre-test work-up.

13. Were uninterpretatable/intermediate test results reported?	Not addressed by Guyatt

14. Were withdrawals from the study explained?	Bias may be introduced if the sample does not comprise consecutive patients presenting with a specific clinical problem


**Additional methodological issues raised, specific to the outcome of 'therapeutic impact'**	• Criteria for establishing therapeutic impact should be formulated a priori.
	• Sufficient clinical information and/or evidence of the effectiveness of available therapies are required to judge whether therapeutic changes will alter patient outcomes

*For the purposes of this comparison the term reference test used in QUADAS is taken as synonymous with the after test in before after studies.

## Abbreviations

CT: Computed tomography; CVA: Cerebrovascular accident; EEG: Electroencephalogram; HTA: Health technology assessment; MRI: Magnetic resonance imaging; NHS: National Health Service; NICE: National Institute for Health and Clinical Excellence; QUADAS: Quality assessment of diagnostic accuracy studies; RCT: Randomised controlled trial; UK: United Kingdom

## Competing interests

The authors declare that they have no competing interests.

## Authors' contributions

CM and CD assessed and modified the QUADAS tool and CM and colleagues applied it to the included studies in the systematic review. CM and CD wrote the manuscript. Both authors contributed to the editing of the paper.

## Pre-publication history

The pre-publication history for this paper can be accessed here:

http://www.biomedcentral.com/1471-2288/9/3/prepub

## Supplementary Material

Additional file 1 **Figure 1. Factors contributing to test impact.** Text at foot – Diagram based on Deeks J. Assessing outcomes following tests. In Price CP, Christenson RH (eds) Evidence-based laboratory medicine. AACC Press, Washington 2007. Figure describing factors contributing to test impact Click here for file

Additional File 2 **Figure 2. Diagnostic before-after study design**. Diagnostic before-after study design Click here for file

Additional File 3 **Figure 3. Diagnostic before-after study specifically for structural neuroimaging in psychosis**. Diagnostic before-after study specifically for structural neuroimaging in psychosis Click here for file
