# Supplementary material for: Quality assessment of diagnostic before-after studies: development of methodology in the context of a systematic review
Source: BMC Med Res Methodol. 2009 Jan 19;9:3. doi: 10.1186/1471-2288-9-3 (PMC2630991; doi:10.1186/1471-2288-9-3)
Supplement: Additional file 1 — Figure 1. Factors contributing to test impact. Text at foot – Diagram based on Deeks J. Assessing outcomes following tests. In Price CP, Christenson RH (eds) Evidence-based laboratory medicine. AACC Press, Washington 2007. Figure describing factors contributing to test impact [file 1471-2288-9-3-S1.doc]

Medical test

Diagnostic information

Diagnostic accuracy

Diagnostic decision

Harms of test and direct effects

Diagnostic yield

Treatment decision

Therapeutic yield

Patient outcomes
