# Supplementary material for: Quality assessment of diagnostic before-after studies: development of methodology in the context of a systematic review
Source: BMC Med Res Methodol. 2009 Jan 19;9:3. doi: 10.1186/1471-2288-9-3 (PMC2630991; doi:10.1186/1471-2288-9-3)
Supplement: Additional File 2 — Figure 2. Diagnostic before-after study design. Diagnostic before-after study design [file 1471-2288-9-3-S2.doc]

Pre-test assessment of clinical problem eg pre-test probability of having disorder

Apply new test

Post-test assessment of clinical problem eg post-test probability of having disorder

Verification of diagnosis using reference standard

Post test assessment of clinical outcomes
