# Supplementary material for: Quality assessment of diagnostic before-after studies: development of methodology in the context of a systematic review
Source: BMC Med Res Methodol. 2009 Jan 19;9:3. doi: 10.1186/1471-2288-9-3 (PMC2630991; doi:10.1186/1471-2288-9-3)
Supplement: Additional File 3 — Figure 3. Diagnostic before-after study specifically for structural neuroimaging in psychosis. Diagnostic before-after study specifically for structural neuroimaging in psychosis [file 1471-2288-9-3-S3.doc]

Pre-test assessment of a, acute psychosis

b, treatment-resistant or deteriorating psychosis

(history, examination, tests)

Post-test assessment of a, acute psychosis

b, treatment-resistant or deteriorating psychosis

Post-test assessment of clinical outcomes

Apply CT/MRI
